# What is the best approach to treat acute migraine in children in the emergency department?

**DOI:** 10.3389/fped.2025.1613580

**Published:** 2025-07-09

**Authors:** Elena Ghirigato, Luisa Zupin, Fulvio Celsi, Valeria Capaci, Alessandro Amaddeo, Giorgio Cozzi

**Affiliations:** ^1^ Clinical Department of Medical Surgical and Health Science, University of Trieste, Trieste, Italy; ^2^ Institute for Maternal and Child Health, IRCCS Burlo Garofolo, Trieste, Italy

**Keywords:** acute migraine, emergency deparment, children, analgesic drugs, acute headache

## Abstract

Migraine is a prevalent condition in children and adolescents, often presenting with severe symptoms that prompt visits to the emergency department (ED). This study aim was to evaluate which is the best approach for treating acute severe migraine in a pediatric ED setting by reviewing randomized controlled trials (RCTs). A systematic literature search was performed and identified 169 articles, of which six met the inclusion criteria, focusing on pediatric patients treated in the ED. The studies reviewed involved various analgesic regimens, including non-steroidal anti-inflammatory drugs (NSAIDs) like ketorolac, anti-dopaminergic drugs like metoclopramide and prochlorperazine, and other treatments like opioids, propofol, and ropivacaine. Available randomized controlled studies are few and heterogeneous in term of drug employed, and do not allow us to directly compare the studies and to identify the best treatment in the emergency department setting. Dopamine antagonists, with or without ketorolac, seems to be the best approach for acute severe migraine in adolescents presenting to the ED.

## Introduction

According to the International Classification of Headache Disorders, migraine is defined as a clinical syndrome characterized by recurrent headache attacks lasting 4–72 h. Typically, the headache is unilateral in location, with moderate or severe intensity, and a pulsating quality. It can be worsened by routine physical activities and may be associated with nausea/vomiting and/or photophobia and phonophobia (1).

Migraine can also be preceded or accompanied by transient focal neurological symptoms, generally referred to as aura. Aura symptoms can be sensory or motor, with visual disturbances being the most common type of aura. In children, migraine often presents with atypical features compared to adults, such as bilateral frontal localization, shorter attack duration, and a frequent association with nausea (2). Another type of migraine manifestation in children is represented by episodic syndromes, including cyclic vomiting syndrome, abdominal migraine, benign paroxysmal torticollis, and benign paroxysmal vertigo. These episodic syndromes, also known as migraine equivalents, are periodic or paroxysmal manifestations that may be associated with migraine or considered prognostic indicators of future migraine development (3).

The pathophysiology of migraine is not yet fully understood, but the trigeminovascular pathway and sensitization of peripheral trigeminovascular neurons are believed to play a critical role in nociception (4).

Additionally, migraine is increasingly regarded as a network disorder involving various cortical, subcortical, and brainstem regions. The hypothalamus acts as a pain facilitator, particularly during the initiation phase, potentially explaining the cyclic nature of the disorder. The thalamus contributes to central sensitization and photophobia, while the cortex is implicated in migraine genesis. Indeed, patients exhibit differences in brain structure, excitability, and functional connectivity (5).

Migraine is a common condition in children and adolescents, with an estimated prevalence of 7%–9.1%, which increases with age, from 5% among children aged 5–10 years to around 15% among teenagers (6, 7).

Some children with chronic migraine may suffer from comorbid anxiety, depressive mood, and impaired psychological functioning, which can lead to school absenteeism and other forms of functional impairment in their daily lives (8).

Moreover, it is a common reason for seeking evaluation in the emergency department (ED). Often, ED patients present with severe symptoms and have already taken oral analgesics as first-line treatment, but without significant relief. Despite the existence of guidelines for managing acute migraine in the ED, treatment approaches still vary among institutions and emergency physicians, particularly regarding pharmacological analgesic strategies (9–11).

Therefore, the primary aim of this systematic review was to examine what the current practice is in the management of acute severe migraine in a pediatric population, and whether there is a drug that outperforms others.

## Search strategy

A systematic review of the literature was conducted in PubMed (https://pubmed.ncbi.nlm.nih.gov) using the optimized search terms: “migraine” or “headache,” and “adolescent” or “children,” and “emergency” (in Title/Abstract), selecting only randomized controlled trials (RCTs), and on Web of Science (www.webofscience.com), using the terms “migraine” or “headache,” and “adolescent” or “children,” and “emergency” (in Title/Abstract) and trial (in all fields). The search was concluded in April 2024. Title and abstract screening of articles was performed manually by two authors independently (E.G. and L.Z.).

Inclusion criteria were: pediatric patients (0–17 years), access to and treatment at the emergency department (ED), clinical diagnosis of migraine, pharmacologic treatment, English language, and peer-reviewed papers. Exclusion criteria were: adult patients, home administration of therapy and non-pharmacological treatment.

The risk of bias was evaluated according to the NHLBI (National Heart, Lung, and Blood Institute) Study Quality Assessment of Controlled Intervention Studies (available at https://www.nhlbi.nih.gov/health-topics/study-quality-assessment-tools) by two authors (L.Z. and E.G.).

## Results

In the PubMed search 169 articles and in Web of Science 63 articles were initially identified. After carefully reviewing the title and abstract, seven studies were initially selected. However, the study of Maki et al. (12) is a pilot study, aiming at determining sample size and feasibility for a superiority RCT regarding the use of intranasal lidocaine. Therefore, only six articles met the inclusion criteria and were included.

The studies main characteristics are reported in Table 1.

**Table 1:** Randomized controlled trials regarding acute treatment of migraine in children, performed in the emergency department setting.

Citation	Study group	Study type	Outcome	Key results	Comments
Brousseau et al. (14) USA	62 children aged 5–18 years Intervention: IV ketorolac (0.5 mg/kg; maximum 30 mg) vs. IV prochlorperazine (0.15 mg/kg; maximum 10 mg).	Double blind RCT (1b)	A 50% or greater reduction in the FPS score at 60 min.	At 60 min prochlorperazine was more effective than ketorolac (84,8% vs. 55,2%; 95% CI: 8–52)	Dopamine antagonists, such as prochlorperazine, could be used alone in migraine management.
Richer et al. (13) Canada	53 enrolled children aged 5–17 years Intervention: IV metoclopramide (0.2 mg/kg; maximum 10 mg) and placebo (0.9% sodium chloride, maximum 1 ml) vs. IV metoclopramide (0.2 mg/kg) and ketorolac (0.5 mg/kg; maximun 30 mg).	Double blind RCT (1b)	Mean change in pain intensity (measured with VAS and FSP-R scales) after 120 min from drug administration.	No significant difference between metoclopramide + placebo vs. metoclopramide + ketorolac (8 mm; *p* = 0.355, 95% CI: −9 to 25)	Combining metoclopramide with other analgesics, such as ketorolac, might not always lead to superior outcomes compared to using metoclopramide alone.
Tsze et al. (15) USA	59 enrolled children aged 8–17 years Intervention: IN ketorolac (1 mg/kg; maximun 30 mg) vs. IV ketorolac (0.5 mg/kg; maximun 30 mg)	Double blind RCT (1b)	Pain reduction of 50% or greater at 60 min using FSP-R (scored 0–10).	At 60 min pain, ≥ 50% reduction in pain: IN ketorolac 88.9% vs. IV ketorolac 93.1%; mean difference between groups was −4.2% (95% CI: −19.2, 10.8).	Intranasal ketorolac appears to be a viable alternative to intravenous administration, offering similar effectiveness.
Boutin et al. (16) Canada	62 enrolled patients aged 8–17 years Intervention: IN fentanyl 1.5 μg/kg (maximun 100 μg) vs. placebo. All participants received oral ibuprofen 10 mg/kg (maximum dose of 600 mg).	Double blind RCT (1b)	Pain relief at 15 min measured with VAS score. Pain reduction (VAS score) at 30 and 60 min.	Mean difference between groups was 2 mm (13 vs. 11 mm, CI 95%: 7–11 mm). Differences in mean pain scores were similar at 30 (4 mm; CI 95%: −8 to 16 mm) and 60 min (6 mm; CI 95%: −9 to 21 mm).	There is no evidence to support the use of opioids for migraine pain in children.
Sheridan et al. (17) USA	66 patients aged 7–19 years Intervention: Low dose of IV propofol (0.25 mg/kg, every 5 min until VAS <4, maximum five doses) vs. Standard therapy (ST) (IV ketorolac 0.5 mg/kg, IV diphenhydramine 1 mg/kg, and IV metoclopramide 0.1 mg/kg	Open-label RCT (1b)	Percentage of pain reduction Length of stay at ED 24 h rebound headache	Pain reduction: 59% (ST) vs. 51% (LDP); (*p* = 0.34) - No difference in length of stay: 79 min LDP vs. 111 min ST; *p* = 0.09. - Rebound headache: 66.7% (ST) vs. 25.0% (LDP) (*p* = 0.01)	No difference, however propofol was associated with significantly fewer rebound headaches.
Yaeger et al. (18) USA	150 children aged 7 to 17 years Intervention: bilateral cervical paraspinal injections of 0.5% ropivacaine or normal saline solution, or a not blinded group receiving no headache therapy for the first 30 min.	Double blind RCT (Ib)	Pain relief on VAS score at 30 min from injection	At 30 min pain relief 32% ropivacaine vs. 28% normal saline solution (effect difference 4%; 95% CI: −14% to 21%)	No difference. Moreover, in both group only a minority achieved pain relief. This may be explained by the overlap with the pain at the injection site, especially considering the short time interval between the injection and the pain assessment.

FSP-R, Faces Pain scale-revised; VAS, visual analogue scale; RCT, randomized controlled trial; IV, intravenous, IN, intranasal, IM intramuscular, ST standard therapy, LDP low dose propofol.

The classification was conducted according to the Oxford Centre for Evidence-Based Medicine: Levels of Evidence (32).

The analyzed studies included a total of 452 children and adolescents, aged between 5 and 17 years, with a number of enrolled patients in each trial ranging from 53 to 150.

The investigated analgesic regimens included ketorolac among non-steroidal anti-inflammatory drugs (NSAIDs), prochlorperazine and metoclopramide among dopamine antagonist drugs, fentanyl among opioids, propofol and ropivacaine among anesthetics.

Drugs were administered at different dosages and through different routes (intravenous, intranasal, intramuscular). Pain intensity was measured using different pain scales (VAS score, Nine Faces Pain Scale, FPS-R scale), but with comparable scores.

Among drugs the most studied were ketorolac and dopamine antagonists.

In particular, three RCTs focused on the use of ketorolac. One study showed that the use of metoclopramide alone was non-inferior to the combination of ketorolac and metoclopramide (13). Another study found that prochlorperazine was superior to ketorolac, (pain response rate of 84.8% vs. 55.2%) one hour after intravenous administration (14). The third study showed non-inferiority of intranasal ketorolac compared to intravenous ketorolac in decreasing headache pain at 30 and 60 min after administration (15).

The only trial considering opioids, focused on the use of intranasal fentanyl combined with oral ibuprofen, and did not show advantage of the combination over ibuprofen alone (16).

One study showed the same efficacy of low-doses of propofol compared to a combination of ketorolac and dopaming antagonists (17).

The last analysed study showed that paracervical injections of ropivacaine were not superior to placebo in decreasing pain (18).

All the examined studies reported the occurrence of adverse events (AEs), which were always minor (mainly nausea/vomiting, dizziness, restlessness, anxiety), with no studies reporting severe AEs. The work by Sheridan et al. describes transient and self-resolving desaturation in a patient receiving propofol (17). Pain at the injection site was the most common side effect reported in the study focused on the effect of ropivacaine (18).

All the studies were graded as 1b, being RCT studies, and evaluated as low for the risk of bias. However, they are few and heterogeneous in terms of the drugs employed, and do not allow us to directly compare the studies or identify the best treatment in the emergency department setting. Therefore, there is insufficient evidence to make a clinical recommendation for a specific type of intervention (10).

## Discussion

Despite the high prevalence of migraine in the general pediatric population, the number of RCTs performed in the ED setting is very limited, and we found only six RCTs focused on the acute treatment of migraine in children, all conducted in North America and recently published, with the exception of a work date to 2004 (Table 1).

The high level of heterogeneity among the existing studies in terms of medication type, dose, route of administration, limited the possibility for comparisons and did not allow to perform a meta-analysis.

Nevertheless, the primary endpoints and study population were quite similar. Headache pain relief, expressed as a reduction in self-reported pain intensity, was the primary endpoint for the majority of the studies and it was assessed at different time points after drug administration.

Notably, ketorolac was the only NSAIDs employed in the trials performed in the ED setting, and data regarding its efficacy are still limited and mixed.

Indeed, its efficacy was inferior to prochlorperazine (14), and its combination with metoclopramide did not result in an improvement in pain score respect to metoclopramide alone (13). Moreover, IV and IN ketorolac route of administration were compared by Tsze et al. (15). The results may suggest that a non-invasive approach may have the same efficacy and may be preferable in some situations. Indeed, considering the pediatric patients where the IV access may be a painful and distressing procedure itself, the use of IN may be easily accepted by patients and parents.

Finally, IV ketorolac was used in combination with diphenhydramine and metoclopramide (defined as standard therapy) and compared to IV propofol (17). The pain reduction is similar between the two groups, with standard therapy performing slightly better to propofol, although it is associated more frequently with 24 h rebound headache, a possible complication due to migraine medication overuse.

On the other hand, commonly used drugs such as acetaminophen, ibuprofen (NSAIDS), triptans, serotonin receptor agonists (19–24), all common drugs employed to treated migraine, were not yet tested in RCT in ED setting.

As previously reported, a group of drugs successfully used in the acute treatment of migraine is antiemetics, particularly antidopaminergic drugs, including prochlorperazine and metoclopramide, which shown a good efficacy (12, 13).

Conversely, there are no studies investigating the efficacy of ondansetron, an antiemetic belonging to the antiserotoninergics class and widely used in pediatrics departments (25, 26).

Opioids are increasingly less used in clinical practice for migraine. Studies have shown that opioids like hydromorphone and tramadol are not superior to standard treatment for migraines, and their use is not recommended (27, 28). The lack of response to conventional analgesic therapy should lead the clinician to consider somatoform pain rather than migraine. Additionally, the only pediatric study comparing intranasal fentanyl to ibuprofen found no superiority of the opioids, further discouraging its use in clinical practice (16).

The last study employing paracervical injections of ropivacaine observed similar effect compared to placebo, and low level of pain relief in both groups (about 30%) at 30′ (18).

Prior to our study, the only systematic review exploring migraine ED treatment in children was conducted in 2008 by Bailey et al. (29). However, their review included only a single RCT study (14), highlighting the limited scope of prior research in this area.

Moreover, most RCT studies on migraine treatment have not been conducted in ED instead, patients were tipically recruited in outpatient clinics and the treatment self-administered at home. Translating the results of these studies to the ED setting is challenging because the severity and duration of symptoms in the ED are often greater. In contrast, outpatient studies usually provide the administration of the medication at the earliest onset of symptoms, which may not fully reflect the acute conditions seen in the ED (30).

It often takes a significant amount of time to adapt new treatment options from adults to children. Indeed, previously works have shown that medication effective in adults may sometimes cause adverse effect in children. Additionally, the placebo effect has occasionally outperformed pharmacological treatments in migraine studies, posing significant challenges to assessing the efficacy of migraine drugs (31).

In the present work, due to the limited number of RCTs available and the high heterogeneity of the analyzed studies, we were not able to identify the best pharmacological treatment approach for acute severe migraine in the ED setting.

So further studies are strongly needed to clarify drugs’ efficacy and standardize management.

## Conclusions

Acute migraine attacks are a common reason for children and adolescents to seek evaluation in the emergency department (ED). Available randomized controlled studies are few and heterogeneous in term of drug employed, and do not allow us to directly compare the studies and to identify the best treatment in the emergency department setting. Dopamine antagonists, with or without ketorolac, seems to be the best approach for acute severe migraine in adolescents presenting to the ED (Grade D) (32).
